# Functional Consequences of GPCR Heterodimerization: GPCRs as Allosteric Modulators

**DOI:** 10.3390/ph4030509

**Published:** 2011-03-14

**Authors:** Karla K.V. Haack, Nael A. McCarty

**Affiliations:** 1 Department of Cellular and Integrative Physiology, University of Nebraska Medical Center, 985850 Nebraska Medical Center, Omaha, NE 68198, USA; E-Mail: kvincent@unmc.edu (K.H.); 2 Department of Pediatrics, Emory University School of Medicine, and Children's Healthcare of Atlanta Center for Cystic Fibrosis Research, 2015 Uppergate Drive, Atlanta, GA 30322, USA

**Keywords:** GPCR, heterodimer, allosteric modulator

## Abstract

G Protein Coupled Receptors (GPCRs) represent the largest family of membrane proteins in the human genome, are the targets of approximately 25% of all marketed pharmaceuticals, and the focus of intensive research worldwide given that this superfamily of receptors is as varied in function as it is ubiquitously expressed among all cell types. Increasing evidence has shown that the classical two part model of GPCR signaling (one GPCR, one type of heterotrimeric G protein) is grossly oversimplified as many GPCRs can couple to more than one type of G protein, each subunit of the heterotrimeric G protein can activate different downstream effectors, and, surprisingly, other GPCRs can affect receptor behavior in G protein-independent ways. The concept of GPCR heterodimerization, or the physical association of two different types of GPCRs, presents an unexpected mechanism for GPCR regulation and function, and provides a novel target for pharmaceuticals. Here we present a synopsis of the functional consequences of GPCR heterodimerization in both *in vitro* and *in vivo* studies, focusing on the concept of GPCRs as allosteric modulators. Typically, an allosteric modulator is a ligand or molecule that alters a receptor's innate functional properties, but here we propose that in the case of GPCR heterodimers, it is the physical coupling of two receptors that leads to changes in cognate receptor signaling.

## 1. Introduction

Given that of the 266 human targets for approved drugs, 27% correspond to family A GPCRs [1], it is not surprising that over the last fifteen years extensive efforts have been devoted to refining existing pharmaceuticals, and identifying new drugs that target and modulate GPCRs. This endeavor was further complicated by the discovery that different GPCRs were capable of physically associating or heterodimerizing, often changing the conventional signaling pathway of a given receptor. Receptor heterodimerization can have substantial consequences on the binding of ligands, as discussed recently by Birdsall [2]. Here, we will provide examples of GPCR heterodimerization and the functional consequences thereof within the framework of allosteric modulation, focusing more heavily on the consequences of heterodimerization on receptor function downstream of ligand binding. We propose that instead of solely investigating the consequences of GPCR-GPCR interaction in terms of changes in signaling or trafficking of a single receptor, one should also consider the physical association with a second receptor as a potential modulator of GPCR activity.

### 1.1. Allosteric Modulation of GPCRs

**Figure 1:**
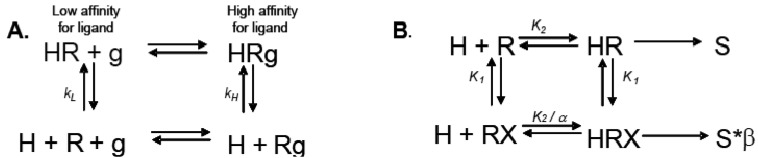
Simple and allosteric ternary complex models. (**A**). The simplest ternary complex model as an adaptation of DeLean *et al.*, taking into account the binding of a hormone or ligand H, to the receptor R, and the modulation by the G protein, g [3]. k_L_ and k_H_, rate constants for the binding of ligand for the low affinity and high affinity state, respectively; (**B**). An allosteric ternary complex model, taking into account the binding at two sites—a site for binding of the orthosteric ligand H, which leads to the relevant stimulus S, and a site for binding of an allosteric modulator, X. The allosteric modulator in the presence of H leads to a modified stimulus, S*β, accounting for the observed changes in response to H in the presence of X. *K_1_* represents the equilibrium constant for binding X, assumed, for the moment, to be the same whether R is also bound by ligand H. *K_2_* represents the equilibrium constant between the orthosteric ligand-free and ligand-bound states. α represents the cooperativity factor, influencing whether there is change of ligand affinity; α > 1 implies a positive cooperativity whereas α < 1 implies negative cooperativity. β represents the change in response to the orthosteric ligand.

Allosteric modulation of GPCRs is a topic of great interest, and has been the subject of a substantial body of literature; unfortunately, due to space limitations, we are unable to reference every paper and, instead, will focus only on seminal studies that are relevant to the concept of modulation of one receptor by another. Early observations made from agonist binding analysis of many GPCRs including the beta-2 adrenoceptor (β_2_AR), muscarinic receptors, dopamine receptors and glucagon receptors all demonstrated the existence of both high and low affinity states of a GPCR. Thus, the simplistic binary model of inactive receptor R → active receptor R* would not work as a sufficient model for describing GPCR signaling. Keeping in mind this notion of a receptor existing in more than one conformation, the shift from high to low affinity states is likely related to increases in second messenger activity leading to feedback onto the receptor itself [3]. The Lefkowitz group first suggested that the signaling cascade of the β_2_AR leading to increases in adenylyl cyclase (AC) activity is the result of a ternary complex that reflects the state of the receptor, R, the ligand or hormone, H, and an additional membrane component (in this instance, the G protein), g. Ligand H can bind saturably and reversibly to the receptor R, and the rate of the reaction is driven by the concentration of the ligand, the equilibrium dissociation constants, and the additional component g (Figure 1A) [3].

**Figure 2:**
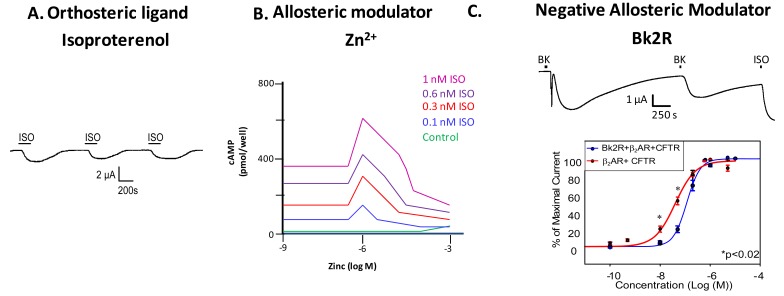
Modulators of β_2_AR. (**A**). Stimulation with isoproterenol (ISO) leads to activation of the cognate signaling pathway; (**B**). Increasing concentrations of Zn^2+^ initially have a positive allosteric effect on ISO-mediated activation of β_2_AR, which then becomes inhibitory [7]; (**C**). B2R is a negative allosteric modulator of β_2_AR, as it is able to activate β_2_AR on its own (top), while having a negative allosteric effect on β_2_AR cognate signaling in response to the selective agonist terbutaline (bottom). Data in parts A and C are from [7].

Just as the canonical example of hemoglobin illustrated that proteins often have more than one binding site, and that these sites can work cooperatively (that binding at one site has influence on the binding at other sites), most GPCRs also have more than one ligand binding site. Most GPCRs possess an orthosteric site where the endogenous ligand binds, and an allosteric site, where another ligand or molecule can bind to modulate receptor activity. Binding of an allosteric modulator can influence receptor signaling and behavior in either a positive or negative direction. All GPCRs have at least one allosteric site; binding of G protein incurs allostery in the most general sense of the term, and can shift the affinity for the orthosteric ligand. Recent studies have shown that many GPCRs bear one or more allosteric sites in addition to the G protein binding site. Allosteric modulators of GPCRs are defined as ligands that bind to an allosteric site on the GPCR to modulate the binding and/or signaling properties of the orthosteric site [4]. Allosteric modulators can increase or decrease the propensity of the receptor to bind the orthosteric ligand by stabilizing the active or the inactive state of the receptor, or by directly altering ligand selectivity. In addition to engineered ligands that serve as allosteric modulators of GPCRs, naturally occurring molecules at physiological concentrations such as ions (as in the case of zinc modulation of β_2_AR) or small proteins (heparin binding to the neurokinin NK1 receptor) also serve as allosteric modulators of GPCRs (Figures 2A, B) [5,6]. GPCRs can also be modulated by other GPCRs (Figure 2C); we will discuss this in depth in a later section.

### 1.2. Types of Allosteric Modulators

Examination of GPCR ligand binding and signaling within the framework of allostery requires further definition of different types of ligands that bind a GPCR. An agonist is a ligand that binds to the orthosteric site to shift the equilibrium to the receptor being in an active conformation (Figure 2A), inverse agonists shift the equilibrium towards the receptor's inactive state, decreasing the basal/constitutive receptor activity, and an antagonist prevents other ligands from binding without a shift in equilibrium. For example, β_2_AR binding of isoproterenol (ISO, in Figure 2A, or H, in Figure 1) would lead to one stimulus (S). Figure 2A is a representative recording of currents elicited in a *Xenopus* oocyte expressing β_2_AR and the Cystic Fibrosis Transmembrane Conductance Regulator (CFTR). When isoproterenol, a non-selective β_2_AR agonist, signals through its cognate G protein leading to subsequent increases in cAMP, PKA phosphorylates CFTR, and opens the channel. Hence, CFTR can be used to monitor the activity of the β_2_AR.

A molecule that binds at the allosteric site does not necessarily require any agonist or inverse agonist functionality of its own, only that it be able to shift the equilibrium of the receptor towards a more active or inactive state by stabilizing or destabilizing a certain receptor conformation. A positive allosteric modulator is a molecule that increases the receptor's affinity or efficacy at binding its ligand at the orthosteric site, or the receptor's ability to enter the active conformation, and a negative allosteric modulator would do the opposite. For example, cinacalcet, a positive allosteric modulator of the Class C Calcium Sensing Receptor, changes the receptor's sensitivity to circulating calcium, thereby being beneficial in disease states such as hyperparathyroidism in which there is a calcium sensing receptor deficiency [8]. Maraviroc, a negative allosteric modulator of the Class A CCR5 chemokine receptor, has been shown to block CCR5-mediated entry of HIV into cells [8]. Additionally, allosteric modulators can possess “pharmacological silence”, as in the case of M-5MPEP, an allosteric ligand for the MGluR5 (Metabotropic Glutamate receptor 5) receptor. M-5MPEP does not inactivate or activate MGluR5, but it partially blocks the response upon agonist binding, leading to partial mGluR5 inhibition [9].

Ions can also modulate GPCRs. For example, zinc has been shown to alter the ligand binding properties of a few different GPCRs at physiologically relevant concentrations (Figure 2B) [10]. Binding of either an agonist or an antagonist to 5-hydroxytryptamine/serotonin receptor 1A (5HT1A-R) is completely prevented by zinc [11]. In the continued presence of zinc, D1 and D2 dopamine receptors do not lose all ligand binding properties like the 5HT1A-R does, but they do have decreased affinity for ligand [12]. Referring back to our example of the β_2_AR in Figure 1B and Figure 2B, addition of zinc (X in Figure 1B) would shift the equilibrium constant such that binding of ISO would lead to a modified stimulus (S*β) compared to the stimulus S in the absence of the allosteric modulator.

We propose that a second GPCR can also act as an allosteric modulator of a GPCR. In the case of the β_2_AR, our previous work has shown that both *in vitro* and *in vivo*, binding of bradykinin (BK) to B2R transactivates β_2_AR [7]. Control experiments show that this transactivation is second messenger independent, and is a result of conformational changes in both receptors [7]. Figure 2C shows that upon coexpression of B2R and β_2_AR, stimulation with BK leads to activation of both a calcium activated chloride channel (as indicated by the sharp current spike) and CFTR (as indicated by the slow current wave); the former is via B2R-mediated activation of Gαq signaling, and the latter is via heterodimer-mediated activation of Gαs signaling [7]. Stimulation with BK a second time also leads to β_2_AR-mediated CFTR opening. These results show that B2R is able to transactivate β_2_AR. Other results suggest that B2R is a negative allosteric modulator of β_2_AR, as shown in the concentration response curve in the bottom panel of Figure 2C. In this case, the concentration response curve generated in *Xenopus* oocytes in response to increasing concentrations of terbutaline, a selective β_2_AR agonist, exhibited a rightward shift in cells also expressing B2R, indicating that B2R dampens the ability of β_2_AR to signal through the Gαs pathway. Because we hypothesize that one GPCR can modulate another, it is important to review the consequences of GPCR/GPCR interaction.

## 2. GPCR Dimerization: A Brief History and Introduction

Until recently, it was thought that two components make up the classical GPCR signaling pathway: the monomeric GPCR and the heterotrimeric G proteins with which it associates. Increasing evidence has shown that this two part model is grossly oversimplified as many GPCRs couple to more than one type of G protein, each subunit of the heterotrimeric G protein can activate different downstream effectors, many modulators exist to alter the signaling of the GPCR, and GPCRs even can alter other effectors in G protein-independent ways. This complexity is further entangled by the emergence of the concept of GPCR dimerization. In 1978, Watanabe and coworkers discovered that methacholine, a non-selective muscarinic receptor agonist, can restore the affinity of the β_2_AR for its agonist isoproterenol in the presence of GTP [13]. This result was later hypothesized to be due to receptor-receptor interaction [14], referred to as dimerization. For our purposes here, dimerization is defined as the physical association of two GPCRs. The possibility of two molecules of the same GPCR being physically coupled (homodimerization) or two different GPCRs being coupled (heterodimerization), presents an unexpected mechanism for GPCR regulation and function. Given the number of other ligand binding proteins that form dimers in order to function, including receptor tyrosine kinases and intracellular steroid receptors, dimerization of GPCRs seemed like a feasible possibility.

The first studies to investigate GPCR dimers used radiation inactivation or target size analysis to study the differences in sizes of molecular species by using high energy particles to disrupt polypeptides, thereby identifying the molecular weight of singular components. This technique was used to illustrate that β_2_AR, opioid receptors (OPRs), muscarinic receptors, Gonadotropin releasing hormone receptors, thyrotropin receptors, and dopamine D1 receptors all formed homodimers [15-17]. The advantages of this technique originally were that the difficulty of both solubilizing and having a great enough yield of receptor membrane protein to detect via other biochemical methods were not limitations to the detection of oligomeric species [18,19].

Although additional methods to study GPCR dimers have grown in the last decade, given the difficulty with resolving endogenous membrane-spanning proteins that are not expressed in relative abundance, most studies rely on transient transfection of cDNA constructs encoding receptors and pharmacological methods such as radioligand competition binding studies to collect indirect evidence for dimerization. These methods are used in conjunction with biochemical methods such as co-immunoprecipitation and bioluminescence or Förster resonance energy transfer (BRET, FRET). Recently, a number of papers have also used bioinformatics and modeling to predict dimer interfaces [20-23]. Excitingly, recent work by Wu and others have illustrated via crystallography that GPCRs can exist as homodimers; the CXCR4 homodimer was crystallized at a resolution of 2.5–3.2 angstroms [24]. All of these techniques have the potential for artifact, so there has been persistent skepticism in proving GPCR dimerization as a verifiable phenomenon. This, in turn, requires that new dimer pairs that are discovered must be found to dimerize both in heterologous and endogenous expression systems. Table 1 provides a representation of a small number of GPCRs that have been shown to heterodimerize either *in vitro*, *in vivo*, or both [1,3,4,25-33].

The canonical examples of GPCR dimerization are the Class C GABA_B_R1 and GABA_B_R2 receptors and the umami and sweet taste receptors [36]. GABA_B_R1 and GABA_B_R2, both Gαi coupled proteins, form naturally occurring obligate dimers meaning that in order to function as a receptor, they must dimerize. This pair was identified in transiently transfected HEK cells by the finding that when either GABA_B_R1 or GABA_B_R2 were expressed singly, the response to ligand was minimal; however, cotransfection robustly increased the response to GABA [36]. These experiments were then repeated in *Xenopus* oocytes injected with cRNAs encoding these receptors and G-protein mediated inwardly rectifying potassium (GIRK) channels to provide a measure of receptor activity [37]. Colocalization and physical association of GABA_B_R1 and GABA_B_R2 were confirmed using immunoprecipitation and confocal microscopy in transiently transfected HEK cells as well as *in situ* hybridization and radiolabeling in rat brains [36]. Interestingly, this seminal work is one of the only examples where GPCR dimerization was studied by electrophysiological methods and uses both heterologous and endogenous expression systems.

Other canonical examples of GPCR heterodimers are the Class C Taste receptors T1R1, T1R2, and T1R3. Detection of sweet taste is mediated by of the T1R2-T1R3 heterodimer, and detection of umami taste is mediated by the T1R1-T1R3 heterodimer. The selectivity of these heterodimers lies in the venus flytrap domain (VFD), where the active site for ligand binding is located. Chimeric experiments have shown that swapping of the VFDs switches the ligand selectivity entirely [38].

A number of studies have examined other examples of GPCR heterodimerization both *in vitro* and *in vivo*, including those summarized in Table 1. Although it is fascinating to examine the physical association of two GPCRs, the potential functionality of that interaction remains the more pertinent question.

## 3. Functional Consequences of GPCR Dimerization

**Figure 3:**
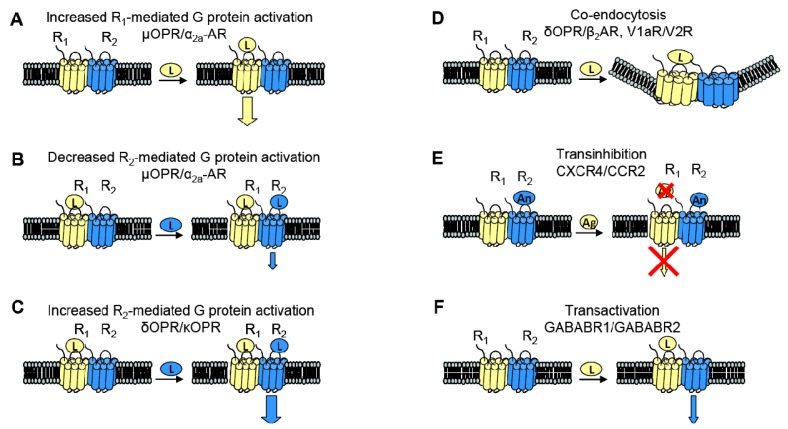
Functional consequences of GPCR heterodimerization. (**A**). As in the case of the μOPR/ a_2a_-AR pair, coexpression of a second receptor can lead to increased G protein activation by the stimulated receptor; (**B**). In the presence of agonists of both receptors, there can be dampened response to a receptor's cognate ligand in the presence of a second receptor as compared to when each receptor is expressed singly; (**C**). Conversely, the addition of a second receptor can increase G protein activation in response to a receptor's cognate ligand; (**D**). Heterodimerization with a second receptor can also change receptor trafficking. For example, when V1aR and V2R are coexpressed, stimulation of either V1aR or V2R leads to co-endocytosis of both receptors; (**E**). As in the case of CXCR4/CCR2 heterodimers, addition of an antagonist against one of the two receptors prevents the other receptor from binding agonist and/or activating, leading to transinhibition; (**F**). Alternatively, one receptor's ligand can lead to transactivation of the second receptor's signaling pathway as opposed to activating R1's cognate pathway.

When examining the interaction between two receptors, potential changes in receptor behavior can include changes in pharmacology, signaling and trafficking. Figure 3 summarizes some of the functional consequences of heterodimerization outlined here.

### 3.1. Changes in Pharmacology

GPCR heterodimerization can lead to changes in a cognate receptor's ability to signal upon ligand binding. Given our previous definition of an allosteric modulator (an entity that binds a receptor at a site other than the orthosteric site thereby changing the properties of that receptor), then GPCRs can also serve as allosteric modulators of one another. In the specific examples detailed below, coexpression of a second receptor, R_2_, changes the properties of the first receptor, R1, thereby altering the response to R1's cognate ligand. Thus, R_2_ is an allosteric modulator of R1 (Figure 3).

The interaction of the family of Gαi-coupled OPRs is a classical example of GPCR heterodimerization leading to changes in pharmacology. It has been shown that κOPR heterodimerizes with δOPR but not μOPR [32]. Although the κOPR/ δOPR heterodimer does not exhibit altered selectivity for either receptor's specific agonist or antagonist, the heterodimer can bind partial agonists for either receptor with increased affinity (Figure 3A). Heterodimerization of these two OPRs also leads to enhanced agonism in the presence of both ligands; addition of the δOPR agonist DPDPE in the continued presence of the κOPR agonist U69563 increases binding affinity and receptor activation (Figure 3C). This synergy is also seen in the presence of both receptor's antagonists, suggesting the presentation of a novel ligand binding pocket in the heterodimer [39]. This κOPR/ δOPR heterodimer was later found to exist *in vivo* because the ligand 6′GNTI, an agonist that has tissue-specific analgesic effects, selectively targets the heterodimer; in contrast, this ligand is able to target the κOPR expressed singly at a very low efficacy and targets δOPR expressed singly not at all [27].

One GPCR in a heterodimer also can have an effect on the other receptor's cognate signaling pathways. The α_2A_-AR and μOPR, both Gαi coupled receptors, when coexpressed in HEK cells were shown to heterodimerize by FRET measurements. Formation of the heterodimer enhances μOPR signaling when stimulated with morphine only, but simultaneous addition of an α_2A_-AR agonist greatly decreased the μOPR signaling response, thus representing both a change in pharmacology and in signaling (Figure 3B). In the inverse experiments, there was also a dampening of norepinephrine-mediated G protein activation in the continuing presence of morphine, but not to the same extent [40].

### 3.2. Transinhbition

Given the aforementioned example of α_2A_-AR and μOPR heterodimers, it is now clear that heterodimerization also can lead to transinhibition of either R_1_ or R_2_ upon addition of antagonist against one of the two receptors (Figure 3E). The Gi-coupled chemokine receptors CCR2 and CXCR4 heterodimerize, and are well studied because of their role in Human Immunodeficiency Virus (HIV) entry into cells and in other inflammatory diseases (CCR2 is expressed on the surface of T lymphocytes and is involved in the recruitment of monocytes to atherosclerotic lesions). Antagonists against either CCR2 or CXCR4 transinhibit the other receptor, preventing binding of chemokines to the opposite receptor in both leukocytes and heterologous cell lines. The CCR2/CCR5 heterodimer also exhibits the same transinhibition [41].

### 3.3. Altered Trafficking

Heterodimerization also can alter trafficking of either receptor compared to when they are expressed singly. For example, when coexpressed in HEK293 cells, V1a and V2 vasopressin receptors can heterodimerize. Although both receptors associate with β-arrestin, V1aR when singly expressed rapidly dissociates from β-arrestin in order to be rapidly recycled to the plasma membrane whereas V2R singly expressed remains stably associated with β-arrestin, thereby accumulating intracellularly. The V1aR/V2R heterodimer followed the V2R endocytic/recycling pattern when stimulated with a nonselective agonist. Selective agonism of the V1aR led to the co-endocytosis of the V2R, suggesting that the heterodimer is influenced by the activated receptor (Figure 3D) [28]. Similarly, cotransfection of β_2_AR, a Gαs coupled receptor, and δOPR, a Gαi coupled receptor, in CHO and HEK cells led to heterodimerization. The heterodimer underwent both isoproterenol-mediated and opioid-mediated endocytosis (Figure 3D). Interestingly, the δOPR/ β_2_AR heterodimer did not exhibit any changes in ligand selectivity compared to each cognate receptor [29]. As mentioned previously, α_1B_-AR and α_1D_-AR also heterodimerize; not only does this interaction change ligand binding characteristics as discussed above, but co-expression of α_1D_-AR greatly increases the trafficking of α_1B_-AR to the cell surface. Without α_1D_-AR present, only negligible amounts of α_1B_-AR are expressed at the cell surface [31].

Despite the increasing amount of knowledge about GPCRs, and the likelihood that they often exist as homo- or heterodimers, there is still a gap in the number of dimer pairs that have been shown to interact in both heterologous *and* endogenous systems (Table 1). Because transfection into a cultured cell line typically creates overexpression of a given protein, it is often suspected that the apparent dimerization and functional consequences are artifacts of non-physiological conditions. Furthermore, methods such as BRET and FRET, often used to study heterodimerization, assume that the introduction of the fluorescent tags attached to the proteins of interest does not induce any false positive interaction. A recent paper from the Sheikh group shows in three separate commonly used cell lines that previously published data on the Angiotensin type II receptor (ATR1)/B2R heterodimer are the result of artifact [42]. Thus, when examining potential interactions, both physical and functional, between two GPCRs, it is of utmost importance to be thorough in technique and cell type using both heterologous and endogenous expression systems to lend credence to statements regarding a putative heterodimer pair.

### 3.4. Allosteric Modulation: Transactivation

Two sets of studies have shown that interactions between two GPCRs also can lead to transactivation, where the ligand-induced conformational change in the R1 receptor leads to activation of the R2 receptor (Figure 3F). The first was the recognition that GABA_B_R1 and GABA_B_R2 must heterodimerize to form a functional receptor. In this case, GABA_B_R1 can bind ligand but cannot activate signaling, while GABA_B_R2 cannot bind ligand, but can activate Gαi-mediated signaling. Hence, binding of ligand to GABA_B_R1 is thought to induce the adoption of an activated conformation by GABA_B_R2. Our own work demonstrated that upon binding BK, B2R is able to transactivate β_2_AR (Figure 2C, top). Additionally, coexpression of B2R is sufficient to hinder β_2_AR's ability to respond to its selective ligand (Figure 2C, bottom). Thus, in this case the B2R is a negative allosteric modulator of β_2_AR [7]. We know that B2R and β_2_AR are physically associated in *Xenopus* oocytes, and in these cells, B2R coexpression alters β_2_AR pharmacology as well as activating this receptor directly in the absence of a β_2_AR-selective ligand (Figure 2C, top). Schwartz and Holst define an allosteric modulator as: “a ligand that functions as both an agonist on its own and as an allosteric modulator of the efficacy and/or potency of the orthosteric ligand” [43]. Although B2R is not a ligand, it fits the definition of an allosteric modulator: BK-bound B2R acts as an agonist at β_2_AR, yet B2R negatively affects the sensitivity of β_2_AR to activation by its cognate ligand.

## 4. A New Model of Allosteric Regulation of GPCRs by Receptor Heterodimerization

**Figure 4:**
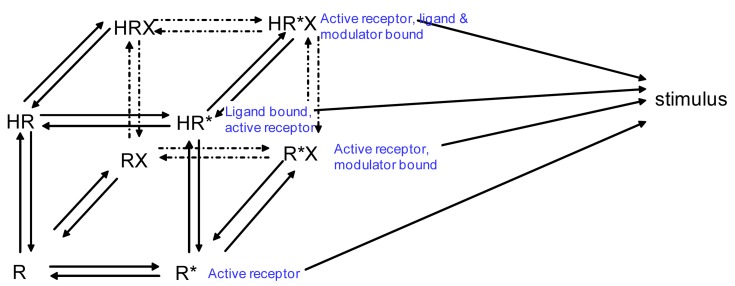
Allosteric two state model. R, inactive receptor; R*, active receptor; H, orthosteric ligand; X, allosteric modulator. Adapted from Hall *et al.* [44].

If we wanted to build a model to represent the interaction of two receptors of a GPCR heterodimer, we must first reflect further on the ternary complex model of GPCRs. A limitation of the previously described ternary complex model is that it cannot factor in the effects on affinity, efficacy, and the number of receptors in the active or the inactive state as determined by specific conformations stabilized by the binding of specific orthosteric or allosteric modulators and their affinity for different receptor states. The allosteric two state model developed by Hall in 2000 factors in the binding of a molecule at an allosteric site on a receptor that switches between inactive and active conformations (Figure 4) [44]. Although Hall's model referred to ligands as allosteric modulators, we focus here on the notion that other receptors also may serve as allosteric modulators of GPCRs via receptor heterodimerization.

**Figure 5:**
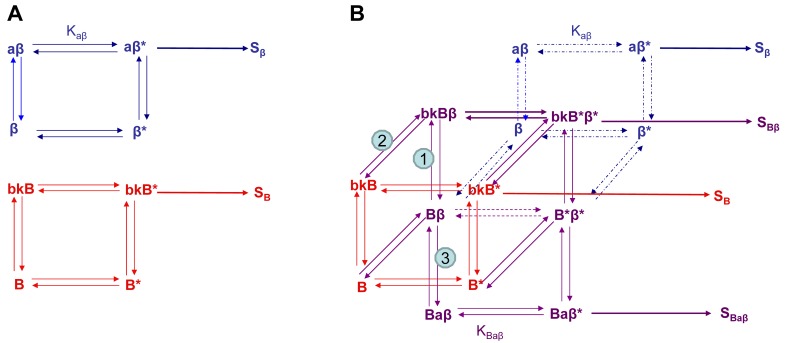
Allosteric model of GPCR heterodimerization, using as an example the interaction of β_2_AR (β, in blue) and B2R (B, in red); ligands are a (isoproterenol) and bk (bradykinin). (**A**). Ternary complex models of both individual receptors; (**B**). The allosteric model of GPCR heterodimerization that includes both B2R, β_2_AR, and B2R/β_2_AR heterodimers. This model includes Bk2R binding bk, which in turn causes both B2R and β_2_AR to adopt active conformations (bkB*β*), leading to activation of both Gαq and Gαs signaling cascades and resulting in stimulus S_Bβ_. Binding of β_2_AR agonist a in the presence of B2R (Baβ*) only activates β_2_AR, leading to stimulus S_BAβ_. In this scheme, we assume that ligand a binding to the monomer/homodimer, e.g., β, is not different from its binding to the heterodimer, Bβ a difference in ligand binding also would affect the potency of a. Note that the model does not differ between monomers and homodimers of a given receptor, or the behavior of the bi-liganded heterodimer, in order to avoid overly complicating the scheme.

Using the B2R and β_2_AR as an example of a GPCR heterodimer, we propose a model that takes into account three different pools of receptors: B2R (monomers and homodimers combined), β_2_AR (monomers and homodimers combined), and B2R/β_2_AR heterodimers; monomers and homodimers are treated the same in order to avoid overly complicating the scheme. As shown in Figure 5A, we can first model either B2R (B) or β_2_AR (β) using the DeLean ternary complex model, in which the receptors fluctuating back and forth between inactive and active (*) conformations can bind their ligands bradykinin (bk in this figure) or isoproterenol (a in this figure), respectively, and thus activate their G protein pathway cascades, leading to stimuli S_B_ or S_β_.

Figure 5B takes into account the formation of functional heterodimers. In this model, the B2R/β_2_AR heterodimer has signaling characteristics different from those of either singly expressed receptor. When B2R of the B2R/β_2_AR heterodimer binds BK (bkBβ) (reaction ➀ in Figure 5B) or when the BK-bound B2R heterodimerizes with β_2_AR (reaction ➁), *both* receptors will switch to an active conformation (bkB*β*). This complex will result in activation of both Gαq- and Gαs-coupled signaling pathways resulting in stimulus S_Bβ_. Although this may not be the case for all other GPCR heterodimers, our model can also take into account the negative allosteric effect that B2R coexpression has on β_2_AR signaling. In this case, when agonist a binds β_2_AR of the B2R/β_2_AR heterodimer (reaction ➂), the equilibrium constant, K_Baβ_, is less than K_aβ_, and the resultant stimulus S_Baβ_ is less than S_β_. It is also possible that heterodimerization with B2R alters the binding of agonist to the β_2_AR; this, too, would affect apparent potency. Our model provides a framework within which to consider the different species of receptors (monomers/homodimers and heterodimers), and the effect of the active and inactive conformations each receptor of the heterodimer can occupy.

## 5. Concluding Remarks

Because there is a possibility of two different GPCRs being physically associated, existing drugs with deleterious side effects are now being re-examined for their ability to target more than one GPCR. For example, levodopa, a dopamine precursor capable of crossing the blood-brain barrier used to treat Parkinson's disease, often has a dyskinesia side effect, thought to be from levodopa-induced co-activation of the A_2A_ adenosine receptor [45]. Thus, it has been proposed that administration of an A_2A_ adenosine receptor antagonist in conjunction with levodopa may decrease some of these side effects [46,47]. This may not be unexpected considering that other work has indicated that these two receptors have both physical and functional interaction in the basal ganglia [48,49]. Taken together, it is quite possible that the existence of GPCR heterodimers should be considered when examining side effects of existing drugs and the presence of GPCR heterodimers may also serve as an axis for developing new drugs. Because GPCR ligands are common drug targets, targeting a heterodimer directly for its allostery could enforce or strengthen the effect of an orthosteric agonist, increase pharmacological diversity, and potentially provide stimulation of a target receptor in cases in which the orthosteric agonist is not present [43]. Conversely, if the site of dimerization is known, then targeting this interaction site for allosteric potentiator or inhibitors as a new class of drug therapies or therapeutic antibody therapies could have specific benefits. It is apparent that the complex signaling pathways and the various modes of regulation that GPCRs undergo make them, to paraphrase a recently published article, a “panacea or Pandora's box for novel drug discovery” [21].

## Figures and Tables

**Table 1:** Heterodimerization of GPCRs [7,25,27,33-35].

	β_1_AR	β_2_AR	V2R	dOPR	kOPR	mOPR	SST2a	α2AR	D_2_	CB_1_	Bk2R	ATR1	V1a	5HT2CR	α1aAR	α1bAR	mGluR2	mGluR4
**β_1_AR**																		
**β_2_AR**																		
**V2R**																		
**dOPR**																		
**kOPR**																		
**mOPR**																		
**SST2a**																		
**α2AR**																		
**D_2_**																		
**CB_1_**																		
**Bk2R**																		
**ATR1**																		
**V1a**																		
**5HT2CR**																		
**α1aAR**																		
**α1bAR**																		
**mGluR2**																		
**mGluR4**																		

	**heterodimer in both *in vitro* and *in vivo***
	**heterodimer in heterologous expression system**
	**unknown**
	**do not dimerize**
